# Cross-sectional study of self-reported pain related to temporomandibular disorders and emotional state of medical school faculty and students: Post-COVID-19 pandemic

**DOI:** 10.1371/journal.pone.0308988

**Published:** 2024-08-26

**Authors:** Alessandra Pucci Mantelli Galhardo, Pâmela Ramos Andrade, Luciana Paes de Andrade, Mariluce Anache Anbar Cury, Marcio Katsuyoshi Mukai, Edmund Chada Baracat, José Maria Soares Jr.

**Affiliations:** 1 Faculdade de Medicina da Universidade Anhanguera-Uniderp, Campo Grande, Mato Grosso do Sul, Brasil; 2 Departamento de Obstetrícia e Ginecologia do Hospital das Clínicas da Faculdade de Medicina da Universidade de São Paulo, São Paulo, Brasil; 3 Departamento de Prótese da Faculdade de Odontologia da Universidade de São Paulo, São Paulo, Brasil; Ohio State University, UNITED STATES OF AMERICA

## Abstract

The social isolation imposed by the COVID-19 pandemic interrupted in-person activities, which were immediately followed by adaptations to ensure, for example, the continuity of teaching. This generated emotional impacts on the academic community. Emotional states may trigger or emphasize conditions such as temporomandibular dysfunction (TMD), causing pain and limiting masticatory function. This study aimed to compare the students and the faculty of a medical school first in terms of the TMD-caused pain they experienced during social isolation and reported two months later, according to their recollections, using the TMD-Pain Screener questionnaire. The second basis for comparison was the emotional state generated by social isolation and its connection with TMD symptoms assessed through the Depression, Anxiety and Stress Scale (DASS-21), which considers perceptions at the time of data collection. Both questionnaires were responded to in May 2022 after the end of social isolation. The data were analyzed by the IBM-SPSS software for Windows 22.0 with a 5% level of significance. The results show that the self-reported TMD symptoms were more widespread (p = 0.002) and intense (p = 0.013) among students than among faculty and that all of the former’s DASS-21 domains (depression, anxiety, and stress) were also more strongly evident (p<0.05). Only anxiety was more significant among the faculty (p = 0.027). Both groups pointed to social isolation as an aggravating factor of the symptoms (p<0.05). The conclusion is that the self-reported TMD-caused pain and all DASS-21 domains as experienced during social isolation were stronger and more prevalent among students than among faculty, and that only anxiety was statistically significant among faculty. Also, the emotional states resulting from social isolation may have aggravated TMD-caused pain in both groups.

## Introduction

Mortality due to COVID-19 brought about compulsory changes in lifestyle, education, and interpersonal relationships. As social agglomeration facilitates the occurrence of infections, isolation measures are required to avoid the possibility of aggravating preexisting conditions or of triggering new diseases. An individual’s emotional component should also be considered because of the effects of a pandemic on human beings: insecurity, uncertainty, and a sense of urgency [1].

The social isolation derived from COVID-19 put a stop to in-person classes, forcing educational institutions to offer alternatives (distance learning) both to lectures and to practical courses [2]. The lack of supervised practical activities generated the profile of an insecure undergraduate conscious of unmet needs, despite the possibility of acquiring experience by acting on the front lines during that time [3, 4]. The uncertainties in such a scenario produced high levels of anxiety and depression among medical students [3, 5–7] and the general population as well [8, 9]. The medical students’ expectations alone regarding their academic performance, professional future, educational standards, and the social demands of their chosen career may lead to emotional imbalance [10].

The characteristics of the medical program interfere in the emotional behavior not only of the students but also of the faculty. Student aid, planning, guidance, administrative issues, and research activities make the academic environment a source of stress and anxiety for the faculty [11, 12]. Within the context of the pandemic, stress levels were heightened by the immediate need for adaptation to new technologies to be able to continue teaching [10, 13].

According to the literature, stress, anxiety, and psychological disorders are possible causative factors that start, sustain, or aggravate temporomandibular disorders (TMD) [6, 14, 15]. Such factors may be related to educational level and type of work, since complaints are more frequent among workers with a higher level of education engaged in intellectual activities than among workers involved in manual labor [7, 16]. Hence, it is not surprising that university students and faculty are targets of TMD and exhibit a greater prevalence of the disorder [7, 16]. Among students, the presence of TMD may be related to the specifics of one’s chosen field of study, and it can induce psychological suffering, parafunctional masticatory habits, sleep disorders, and teeth grinding [17–21]. In short, this pathology has a multifactorial origin and is characterized by a masticatory functional limitation and/or pain in the orofacial regions and in the structures associated with the temporomandibular articulation.

Salivary cortisol and anxiety levels are associated with myogenic TMD [22], and thus pandemic social isolation might be regarded as a TMD stressor and perpetuator [23–26]. In the individuals identified with TMD before the COVID-19 pandemic, chronic pain was aggravated by rising anguish, anxiety, and depression due to the pandemic, which proves there is a correlation between psychological issues and TMD symptoms [6, 27–29].

The anxiety and stress generated by social isolation may have been experienced by both the faculty and the students. While the former had to adapt instantly to offer remote classes without sufficient training for this, the students might have felt insecure about not being fully able to assimilate content offered in a remote format and wondered whether they would be sufficiently prepared to face the job market. Therefore, this study aimed to compare the students and the faculty members of a medical school in terms of the following two points: (1) the TMD-caused pain they experienced during social isolation and reported two months thereafter according to their recollections, using the TMD-Pain Screener questionnaire; (2) the emotional state generated by social isolation and its connection with the TMD symptoms of both groups, assessed through the Depression, Anxiety and Stress Scale (DASS-21), which considers perceptions at the time of data collection. Both questionnaires were responded to in May 2022, two months after social isolation came to a close at the end of February.

## Materials and methods

### Study design

This is an analytical cross-sectional observational study conducted in accordance with the guidelines of Strengthening the Reporting of Observational Studies in Epidemiology–STROBE [30].

### Context

The study was carried out at the School of Medicine of Anhanguera-Uniderp University in Campo Grande, MS, Brazil with the participation of students and faculty members. The research project and the free prior and informed consent (FPIC) statement were approved by the institutional research ethics committee (*CEP* Anhanguera-Uniderp) under protocol No. 5.332.154.

### Participants

Fifty-seven faculty members and 73 actively enrolled students participated in the study. The students were recruited from all semesters of the medical school, for it is our understanding that the pandemic interfered in all of the phases of medical training. Entrants in the year 2022 (faculty or students) were excluded from participation because they did not experience the social isolation in the academic environment imposed by the COVID-19 pandemic nor were they exposed to distance learning or teaching. All participants were older than 18.

### Variables

The self-reported TMD-related pain was the main variable that was checked. Assessment was equally made in both groups with the TMD-Pain Screener, a tool for TMD-caused pain that is part of Axis I of Diagnostic Criteria for Temporomandibular Disorders (DC/TMD) and that allows the identification of TMD-related pain through three self-directed questions [7].

The variables connected with psychological states (anxiety, depression, and stress) were analyzed using the Depression, Anxiety and Stress Scale– 21 (DASS-21) in the official version of the Portuguese language [31]. The questionnaire was administered to both students and faculty.

The possible influence of the emotional state resulting from social isolation on TMD-caused pain was evaluated with the following questions: “Do you remember if these symptoms were present at the beginning of the pandemic?”, “If yes, were they worse than now?”, and “Do you believe the pandemic exacerbated the symptoms?”

Considering that TMD is a multifactorial disorder, there might have been confounding factors that were not assessed, such as malocclusions, missing teeth, or parafunctional habits. This study was not conducted in person, precluding a clinical analysis of such factors.

### Collection of variables: Research tool and questionnaires

The data were obtained from questionnaires created using Google Forms and sent to the participants via a link in the Whatsapp^®^ app at the beginning of May. For both the students and the faculty members, the first part of their questionnaires was the FPIC statement, which at the end presented the options to participate or not to participate in the study. It was necessary to click on the acceptance option to be able to proceed to the next part, the questionnaire *per se*. As the choice of this option indicated consent to participate in the study, no actual signature on paper was required. The selection of the nonacceptance option blocked the appearance of the second part of the questionnaire, keeping the candidate from proceeding. The candidates who agreed to take part in the research then identified themselves as faculty members or students and were directed towards the specific questionnaire of their group. Most questions were the same for both the students and the faculty members. Some questions, however, were pertinent to the activities of only one group, such as academic titles, length of stay at the institution (for faculty), and the semester of medical school in which the student was enrolled at the onset of social isolation (for students). This question, in particular, enabled the exclusion of students who entered the medical school in 2022, since they did not experience distance learning. Both questionnaires consisted entirely of multiple-choice questions.

#### Analysis of the pain caused by temporomandibular dysfunction

To assess TMD-related pain, screening for pain was carried out with the TMD Pain Screener [32], which is part of Axis I of Diagnostic Criteria for Temporomandibular Disorders (DC/TMD) [7], a tool made up of two axes: Axis I and Axis II. Axis I, when used in its entirety, provides diagnoses of the main TDM types based on a patient’s clinical examination. This we were unable to perform given the context of social isolation. Instead, we used the TMD Pain Screener, which is part of Axis I. This pain screener only identifies the individual who has pain associated with TMD, and does so through 3 self-directed multiple-choice questions, without actually reaching a diagnosis. The three questions with their possible answers are the following: 1. "In the last 30 days, how long did any pain last in your jaw or temple area on either side? a. No pain; b. Pain comes and goes; c. Pain is always present”; 2. “In the last 30 days, have you had pain or stiffness in your jaw on awakening? a. No; b. Yes”; 3.” In the last 30 days, did the following activities change any pain (that is, made it better or made it worse) in your jaw or temple area on either side? A. Chewing hard or tough food (a. No; b. Yes); B. Opening your mouth or moving your jaw forward or to the side (a. No; b. Yes); C. Jaw habits such as holding teeth together, clenching, grinding, or chewing gum (a. No; b. Yes); D. Other jaw activities such as talking, kissing, or yawning (a. No; b. Yes).”

The answers to question 1, that is, *a*, *b*, *and c*, were worth 0,1, and 2 points, respectively. In the two other questions, each instance of a *no* answer was worth 0, and each instance of a *yes* answer was worth 1. Thus, the highest possible score was 7. A score of 2 or more meant the individual had TMD-related pain.

As intended by the authors and only for the purposes of this research, the participant’s final score was used to categorize the TMD-related pain as follows: a score equal to zero meant absent pain from TMD; 1 or 2 meant a mild degree of pain; 3 to 5 meant moderate pain; and 6 or 7 meant intense pain.

The participants who scored 1 or 2 points were classified as not having TMD-related pain. Nonetheless, they answered the questions concerning the role of social isolation in TMD-related pain as discussed below.

#### Social isolation and TMD-caused pain

In order to determine whether the social isolation and emotional states resulting from the COVID-19 pandemic were possibly connected with TMD-caused pain, the TMD-Pain Screener was administered. Two or three additional questions were also asked. These tried to ascertain whether the painful symptoms arising from TMD were present at the beginning of the pandemic, whether the symptoms at such a time were more or less distressing (retrospective analysis), and whether the participant attributed the exacerbation of the symptoms to the pandemic. The questions were the following: “Do you remember if these symptoms were present at the beginning of the pandemic?”; “If yes, were they worse than now?”; and “Do you believe the pandemic exacerbated the symptoms?”. Each question had only two possible answers: yes or no.

#### Analysis of emotional states

The Depression, Anxiety and Stress Scale ‐ 21 (DASS-21) in the official version of the Portuguese language was used for analyzing psychological states. The DASS-21 questionnaire consists of 21 questions organized into 3 domains: depression, anxiety, and stress. Each domain has 7 specific questions, the answers to which have different values: zero (did not apply to me at all ‐ NEVER), one (applied to me to some degree or sometimes–SOMETIMES), two (applied to me to a considerable degree or a good part of the time–OFTEN) and three (applied to me very much or most of the time–ALMOST ALWAYS). Each domain or subscale yields a final score, which is the sum of the scores of its 7 items multiplied by 2, and it is analyzed according to the cutoff points shown in Table 1.

**Table 1:** Classification of the DASS-21 domains according to the final scores.

	Zero score	Percentile	Depression	Anxiety	Stress
**Normal**	<0.5	0–78	0–9	0–7	0–14
**Mild**	0.5–1.0	78–87	10–13	8–9	15–18
**Moderate**	1.0–2.0	87–95	14–20	10–14	19–25
**Severe**	2.0–3.0	95–98	21–27	15–19	26–33
**Extremely severe**	>3	98–100	28+	20+	34+

^*^Depression, Anxiety, and Stress Scale– 21 (DASS-21), Portuguese language version [31].

### Bias

Social isolation in Campo Grande-MS was decreed in May 2020 and data collection for this study took place in May 2022, two months after classroom lectures were fully reestablished on the first days of March. However, as some of the information (TMD-related pain) that was recalled referred to the time when social distancing measures were implemented, it was subject to a memory bias.

### Sample size

The sample size calculations for faculty members and students alike were made considering conservative samples of finite populations representing the total number of students and faculty members of the institution. Thus, the power of the collected sample was 85%, with a precision of 10.9% for students and 10.6% for faculty members and a confidence level of 95%.

### Statistical methods

Analyses were performed with the IBM-SPSS software for Windows version 22.0 and data tabulation was accomplished with the Microsoft Excel (2010) software. The tests were run with a 5% significance level (p<0.05).

The qualitative characteristics were described for each group using absolute and relative frequencies. The quantitative characteristics were described with summary measures (mean and standard deviation or median and minimum and maximum).

Variables common to both groups (faculty members and students) were related to each group by means of association tests, chi-square tests, or likelihood ratio tests. For the ordinal characteristics, the chi-square trend tests were used. The ordinal qualitative items were compared with the Mann-Whitney tests. The domains of the DASS-21 scale were described and comparisons were made between the groups with the Mann-Whitney tests.

The correlation between age and TMD-caused pain was determined through the Spearman correlation tests, and that between marital status and TMD-caused pain, through the chi-square test.

## Results

The medical school faculty was comprised of a total of 111 members, 33 of whom were not eligible for this study because they joined the school after the period of social isolation. Of the 78 remaining faculty members, only 57 answered the questionnaire.

At the time this research was conducted, there were 447 actively enrolled students, 203 of whom were not eligible because they enrolled when social isolation was over and 84 because they were under 18 years of age. Of the remaining 160 eligible students, only 73 volunteered to answer the questionnaires.

### Descriptive demographic data

Seventy-three students and 57 faculty members, totaling 130 individuals, participated in this study. The demographic characteristics are described below in Table 2.

**Table 2:** Sociodemographic characteristics.

Variables	Groups		Total (n = 130)	p
	Students (n = 73)	Faculty (n = 57)		
**Age (years)**				<0.001^a^
Mean ± SD	24.1 ± 5.9	42.7 ± 10.1	32.3 ± 12.2	
Median (mIn;max)	23 (18;43)	42 (27;77)	30 (18;77)	
**Sex, n (%)**				0.168^b^
Male	12 (16.4)	15 (26.3)	27 (20.8)	
Female	61 (83.6)	42 (73.7)	103 (79.2)	
**Marital status, n (%)**				<0.001^c^
Married	13 (17.8)	45 (78.9)	58 (44.6)	
Single	58 (79.5)	8 (14)	66 (508)	
Others	2 (2.7)	4 (7)	6 (4.6)	
**Academic titles, n (%)**				
Specialization	-	32 (56.1)	-	
Master’s degree	-	14 (24.6)	-	
Doctoral degree	-	10 (19.3)	-	
**Length of stay at the institution, n (%)**				
Less than 3 years	-	19 (33.3)	-	
From 3 to 6 years	-	10 (17.5)	-	
More than 6 years	-	28 (49.1)	-	

min means minimum; max means maximum

n means number of participants

% means relative Frequency

Total corresponds to the total number of research participants, comprising faculty and students

Under marital status, “others” encompasses stable unions, widowhood, or separation from spouse

^a^Statistically significant difference between the groups per Student t test (p<0.001)

^b^Result with no statistical significance per chi-square test

^c^Statistically significant difference between the groups per likelihood ratio test

^d^Result with no statistical significance per the Mann-Whitney test.

Table 2 shows that the students’ mean age was statistically lower than that of the faculty members (p<0.001) and that the distribution of marital status was statistically different between the groups (p<0.001). There was a higher relative frequency of married people among the faculty and a prevalence of their members with specialization (56.1%). Nearly most of the faculty members (49.1%) who participated in this study had over 6 years of commitment to the institution.

### TMD-related pain

The difference in presence and intensity of TMD-related pain between students and faculty was statistically significant (p = 0.002 and p = 0.013, respectively), as shown in Table 3.

**Table 3:** Presence of self-reported TMD-caused pain and degrees of severity among students and faculty.

TMD-pain characteristics	Groups		Total (n = 130)	p
	Students (n = 73)	Faculty (n = 57)		
**TMD-pain, n (%)**				0.002^a^
Absent	29 (39.7)	38 (66.7)	67 (51.5)	
Present	44 (60.3)	19 (33.3)	63 (48.5)	
**Degree of severity, n (%)**				0.013^b^
Absent	29 (39.7)	38 (66.7)	67 (51.5)	
Mild	7 (9.6)	3 (5.3)	10 (7.7)	
Moderate	25 (34.2)	8 (14)	33 (25.4)	
Intense	12 (16.4)	8 (14)	20 (15.4)	

TMD means temporomandibular disorder

n means number of participants

% means relative frequency

Total corresponds to the total number of research participants, comprising faculty and students

Degrees of severity of TMD: mild corresponds to a score equal to or less than 2; moderate corresponds to a score between 3 and 5; intense corresponds to a score between 6 and 7; scores were calculated according to Ohrbach et al. (2014) [7]

^a^Statistically significant result according to the chi-square test (p<0.05)

^b^Statistically significant result according to the Mann-Whitney test.

There was no correlation between age and severity (in degrees) of TMD-caused pain neither among students (p = 0.742; r = 0.04; IC = -0.2–0.3) nor among faculty (p = 0.177; r = -0.1812; IC = -0.4–0.1) according to the Spearman correlation test.

Marital status was not associated with severity (in degrees) of TMD-caused pain neither among students (p = 0.213) nor among faculty (p = 0.282) according to the chi-square test.

### Emotional states: Anxiety, depression, and stress (DASS-21)

A comparison of the mean scores obtained on the DASS-21 domains by students and faculty was significant in all three domains: depression, anxiety, and stress. The data in Table 4 demonstrates this.

**Table 4:** Degrees of severity in each DASS-21 domain of students and faculty.

Variables	Groups		p
	Students (n = 73)	Faculty (n = 57)	
**Depression, n (%)**			P = 0.001^a^
Normal	42 (57.5)	48 (84.2)	
Mild	3 (4.1)	5 (8.8)	
Moderate	12 (16.4)	4 (7)	
Severe	6 (8.2)	0 (0)	
Extremely severe	10 (13.7)	0 (0)	
**Anxiety, n (%)**			
Normal	37 (50.7)	48 (84.2)	p<0.001^a^
Mild	2 (2.7)	2 (3.5)	
Moderate	10 (13.7)	3 (5.3)	
Severe	8 (11)	1 (1.8)	
Extremely severe	16 (21.9)	3 (5.3)	
**Stress, n (%)**			
Normal	40 (54.8)	49 (86)	p<0.001^a^
Mild	4 (5.5)	2 (3.5)	
Moderate	6 (8.2)	5 (8.8)	
Severe	12 (16.4)	0 (0)	
Extremely severe	11 (15.1)	1 (1.8)	

n means number of participants

% means relative frequency

The depression, anxiety, and stress domains, assessed as degrees of severity (normal, mild, moderate, severe, and extremely severe), comprise the Depression, Anxiety and Stress Scale– 21 (DASS-21), officially translated into the Portuguese language [31]

^a^Statistically significant result according to the Mann-Whitney test.

### Link between the TMD-caused pain and emotional states resulting from the COVID-19 social isolation

The results show that anxiety was statistically significant in faculty members identified with TMD-caused pain (p = 0.027). Temporomandibular symptoms were strong at the onset of the pandemic (p = 0.005), and their aggravation, attributed to emotional states brought on by social isolation, revealed a statistically significant association (p<0.05) with it.

As for the students, all DASS-21 domains exhibited higher scores, which were all statistically significant in association with TMD-related pain (p<0.05). The characteristics of the temporomandibular symptoms at the onset of the pandemic and the role the pandemic played in exacerbating the symptoms were found to have a statistically significant association in every student participating in the study (p<0.001) (Table 5).

**Table 5:** Relationship between self-reported TMD-caused pain and emotional states according to faculty and students within the context of the pandemic.

Variable	TMD-pain–Faculty		Total	p	TMD-pain–Students		Total	p
	Absent	Present			Absent	Present		
**Depression**								
Normal	34 (89.5)	14 (73.7)	48 (84.2)	0.106^a^	20 (69)	22 (50)	42 (57.5)	0.036^b^
Mild	3 (7.9)	2 (10.5)	5 (8.8)		3 (10.3)	-	3 (4.1)	
Moderate	1 (2.6)	3 (15.8)	4 (7)		3 (10.3)	9 (20.5)	12 (16.4)	
Severe	-	-	-		2 (6.9)	4 (9.1)	6 (8.2)	
Extremely severe	-	-	-		1 (3.4)	9 (20.5)	10 (13.7)	
**Anxiety**								
Normal	35 (92.1)	13 (68.4)	48 (84.2)	0.027^b^	19 (65.5)	18 (40.9)	37 (50.7)	0.003^b^
Mild	1 (2.6)	1 (5.3)	2 (3.5)		2 (6.9)	-	2 (2.7)	
Moderate	-	3 (15.8)	3 (5.3)		5 (17.2)	5 (11.4)	10 (13.7)	
Severe	-	1 (5.3)	1 (1.8)		2 (6.9)	6 (13.6)	8 (11)	
Extremely severe	2 (5.3)	1 (5.3)	3 (5.3)		1 (3.4)	15 (34.1)	16 (21.9)	
**Stress**				0.068^a^				
Normal	35 (92.1)	14 (73.7)	49 (86)		22 (75.9)	18 (40.9)	40 (54.8)	0.001^b^
Mild	-	2 (10.5)	2 (3.5)		2 (6.9)	2 (4.5)	4 (5.5)	
Moderate	3 (7.9)	2 (10.5)	5 (8.8)		2 (6.9)	4 (9.1)	6 (8.2)	
Severe	-	-	-		3 (10.3)	9 (20.5)	12 (16.4)	
Extremely severe	-	1 (5.3)	1 (1.8)		-	11 (25)	11 (15.1)	
**TMD-pain**								
**Presence of symptoms (onset of the pandemic), n (%)**								
Yes	8 (21.1)	11 (57.9)	19 (33.3)	0.005^c^	-	21 (47.7)	21 (28.8)	<0.001^c^
No	30 (78.9)	8 (42.1)	38 (66.7)		29 (100)	23 (52.3)	52 (71.2)	
**Symptom characteristics (onset of the pandemic), n (%)**								
Better than today	7 (18.4)	10 (52.6)	17 (29.8)	0.001^d^	2 (6.9)	24 (54.5)	26 (35.6)	<0.001^d^
No symptoms	29 (76.3)	5 (26.3)	34 (59.6)		27 (93.1)	11 (25)	38 (52.1)	
Worse than today	2 (5.3)	4 (21.1)	6 (10.5)		-	9 (20.5)	9 (12.3)	
**Did the pandemic exacerbate the symptoms? n (%)**								
Maybe	2 (5.3)	4 (21.1)	6 (10.5)	<0.001^d^	2 (6.9)	15 (34.1)	17 (23.3)	<0.001^d^
No symptoms	28 (73.7)	-	28 (49.1)		26 (89.7)	1 (2.3)	27 (37)	
The pandemic did not aggravate the symptoms	7 (18.4)	9 (47.4)	16 (28.1)		1 (3.4)	12 (27.3)	13 (17.8)	
Yes, for sure	1 (2.6)	6 (31.6)	7 (12.3)		-	16 (36.4)	16 (21.9)	

TMD means temporomandibular dysfunction.

n means number of participants

% means relative frequency

Total corresponds to the total number of faculty and students participating in the study

The depression, anxiety, and stress domains, assessed in terms of degrees of severity (normal, mild, moderate, severe, and extremely severe), comprise the Depression, Anxiety and Stress Scale– 21 (DASS-21), officially translated into the Portuguese language [31]

^a^Result with no statistical significance per the Mann-Whitney test

^b^Result with statistical significance per the Mann-Whitney test

^c^Result with statistical significance per the chi-square test

^d^Result with statistical significance per the likelihood ratio test.

## Discussion

Temporomandibular dysfunction occurs frequently and causes pain and restricted masticatory function. An emotional component is part of its multiple etiology [18, 20, 21]. The COVID-19 pandemic may be an added emotional factor further influencing TMD-pain intensity in students, who are less knowledgeable and more vulnerable to emotional components (anxiety, depression, and stress) than faculty members [20]. This distinction is in line with our results, which demonstrated that all of our students’ DASS-21 domains were strong, while anxiety was the sole prevalent domain among faculty members.

The literature points out that medical students are more prone to mental health disorders mainly as a consequence of several factors: their own and their families’ expectations towards their chosen career; the social pressure they are under relative to their academic performance, professional future, and life inexperience [10]; and the pressure derived from the number of required study hours, which limits social life and leisure, tools that aid in the control of emotional health [12, 20, 22]. Besides, stress may trigger cognitive deficits, signaled by memory loss and difficulty assimilating new information. These deficiencies hamper academic performance and thus sustain the cycle of chronic orofacial pain [19] associated with the emotional states among students.

Among faculty, it is possible that sleep disorders and the burnout syndrome contribute to the manifestation of TMD symptoms, for they are conditions usually encountered in this group [11]. Similarly, as anxiety turned out to be significant, it may have triggered TMD symptoms. Anxiety probably resulted from the immediate need to teach in the distance learning mode, a situation for which not all members felt prepared.

Anxiety, depression, and stress are emotional states that promote TMD symptoms [6, 14, 15, 22, 24] and contribute to the development and maintenance of other types of pain [28]. When confronting an unpredictable situation, the body reacts with a stress response, possibly giving rise to anxiety, particularly in prolonged situations such as that of the COVID-19 pandemic [8, 9, 24]. An overcharged system experiences a high level of sympathetic activity [12] that elicits responses, such as muscle vasoconstriction and increased peripheral vascular resistance, through the release of adrenocortical steroids [14]. For example, high levels of cortisol are present during muscle hyperactivity in myogenic TMD [22], altering the blood circulation of the muscles related to masticatory function and bringing on pain. If the body is not under stress, the prefrontal region regulates thoughts, emotion, and behavior, inhibiting inadequate motor responses. On the other hand, if the body is under stress, the amygdala activates the hypothalamus and brainstem. This activation hinders the regulation of the prefrontal cortex [8, 14], which produces individualized responses according to one’s own (subjective) repertoire, determining the intensity of suffering [6, 15, 22, 24]. It follows that circumstances that prolong stress may intensify anxiety and the pathologies derived from it, such as TMD [8, 29]. Although cortisol levels were not measured in our study, the hormone is related to exacerbation of TMD pain in some studies [8, 29] and may be a target for further studies to justify the effects of social isolation as a stressor.

Social isolation as an etiological agent of stress [8, 24, 29] is based on the fear originating from the possibility of contracting the virus [28], and insecurity as to what is known and can be done to control the SARS-CoV-2 virus (the etiological agent of COVID-19). Besides, concerns about the economic impact of the pandemic, lack of confidence in government institutions to confront the crisis, curtailment of family life, and dissemination of fake news [1] can also generate stress. Such perceptions are individually processed in accordance with personal vulnerabilities and tolerance, and thereby may be viewed as triggers for the development of anxiety [22, 27, 29]. In some cases, there may be physical manifestation of posttraumatic stress impacting musculoskeletal pain as present in TMD [18, 28]. The anxiety possibly brought on by social isolation also exacerbates the symptoms of pain in the masticatory muscles, more so in individuals with a higher level of education [27]. The results of this study show that TMD-caused pain was pronounced at the onset of social isolation for students and faculty alike and that the pandemic period was possibly a potential reason for the aggravation of pain.

Contrariwise, as the pandemic progressed, there were reports about an increasing number of individuals cured of COVID-19. Moreover, there were indications that a healthy lifestyle prior to the pandemic was a positive influence on the outcome of the disease. A possible result was a decrease in stress and the relief of TMD-caused pain during the period of social isolation [8], showing that individual tolerance and human adaptive capacity are influential in the development and maintenance of pathologies. However, it is important to reinforce that specific characteristics of the medical courses and academic environment [20] may have had an impact on individual responses and that improvement in the disorders under analysis would be unlikely during the isolation period of the pandemic [28] in this public. Still, social isolation is not deemed a continuous event, and it may produce transitory symptoms of a sufficient magnitude to exacerbate TMD-caused pain momentarily [24] as found in this cross-sectional study.

Distance learning imposed by social isolation stimulated stress, anxiety, and depression in the students [4, 6, 16, 19, 23, 26], since it produced a sensation of ineptitude to face the job market as health professionals [3]. This may have been partly responsible for the exacerbation of TMD-caused pain in our students, such as found in other studies [6, 18, 20, 25, 31].

As for the faculty, TMD-caused pain was also present, perhaps mostly due to the intense mental tiredness prompted by the need to adapt to remote teaching. Previous literature [2, 13] shows that not being familiar with the technology involved in this teaching approach is a reason for educators to feel stressed. This may also be a factor that explains the presence of anxiety in faculty members, the only DASS-21 domain that was statistically significant for this group. Evidently, there is a need for greater knowledge of such technologies if one is contemplating the possibility of new pandemics [5]. Studies show that the more educated individuals are and the more time they spend working, the more pronounced the TMD symptoms are [17, 27].

This study has a few limitations. The students were assessed as a group with no consideration for the semester of medical school each student was in. Despite the discontinuation of the in-person classes having consequences for all stages of academic learning, it is likely that the students in the final semesters of the medical program, which consist entirely of practical courses, were at a greater disadvantage. Such a drawback translated as a sensation of ineptitude. Another limitation was the small sample size of the study; however, power was approximately 85%. Regardless of these facts, we would like to stress the relevance of our results. Our findings were corroborated by a group of psychologists, the Educational Guidance Group (*Grupo de Orientação Educacional*, GOE). Their objective is to assist students in need of psychological support within our university, and they worked intensively during the pandemic to help our students. Additionally, considering that full resumption of face-to-face classes occurred only at the beginning of March 2022, two months before the data for this study were gathered in May 2022, there may have been an information bias applying to the variables pertinent to the onset of the pandemic. The questions on the TMD-Pain Screener require answers lying within the 30-day period prior to the administration of the questionnaire, and those on DASS-21, within the week prior to data collection. This is how the two tools were built and are found in the validated versions in the literature. These tools were used to collect data after the end of social isolation not within the period of social isolation itself. Nevertheless, the data we obtained could stand as a reflection of what was felt during social isolation. The participants’ feelings were still alive at the time they were checked (May 2022) as their responses led us to believe. Furthermore, we were careful to obtain retrospective data on TMD pain felt during the social isolation period and to establish links between the study variables. Finally, given the social isolation during the COVID-19 pandemic, no actual diagnoses of TMD were made.

There are few studies in the literature addressing the TMD-caused pain in the academic community within a context such as the pandemic. The academic community is an environment with high emotional demands, which may interfere in conditions such as TMD, exacerbating its symptoms.

## Conclusions

Considering only individuals who experienced the COVID-19 social isolation, self-reported TMD-caused pain and all DASS-21 domains (stress, anxiety, and depression) were stronger and more prevalent among students than among faculty. Only anxiety reached a statistically significant level among faculty members. Emotional states resulting from social isolation also seemingly worsened TMD-caused pain in both groups.

## Supporting information

S1 Data (XLSX)

S2 Data (XLSX)
